# Effect of Type and Concentration of Nanoclay on the Mechanical and Physicochemical Properties of Bis-GMA/TTEGDMA Dental Resins

**DOI:** 10.3390/polym12030601

**Published:** 2020-03-06

**Authors:** J. J. Encalada-Alayola, Y. Veranes-Pantoja, J. A. Uribe-Calderón, J. V. Cauich-Rodríguez, J. M. Cervantes-Uc

**Affiliations:** 1 Centro de Investigación Científica de Yucatán, A.C. Unidad de Materiales, Calle 43 No. 130 x 32 y 34, Col. Chuburná de Hidalgo C.P. Mérida 97205, Mexico; juan.encalada@cicy.mx (J.J.E.-A.); jorge.uribe@cicy.mx (J.A.U.-C.); jvcr@cicy.mx (J.V.C.-R.); 2 Centro de Biomateriales, Universidad de La Habana, Avenida Universidad, s/n, e/G y Ronda, C.P. La Habana 10600, Cuba; yayma@biomat.uh.cu

**Keywords:** dental resin composite, montmorillonite, palygorskite

## Abstract

Bis-GMA/TTEGDMA-based resin composites were prepared with two different types of nanoclays: an organically modified laminar clay (Cloisite^®^ 30B, montmorillonite, MMT) and a microfibrous clay (palygorskite, PLG). Their physicochemical and mechanical properties were then determined. Both MMT and PLG nanoclays were added into monomer mixture (1:1 ratio) at different loading levels (0, 2, 4, 6, 8 and 10 wt.%), and the resulting composites were characterized by scanning electron microscopy (SEM), thermogravimetric analysis (TGA), dynamic mechanical analysis (DMA) and mechanical testing (bending and compressive properties). Thermal properties, depth of cure and water absorption were not greatly affected by the type of nanoclay, while the mechanical properties of dental resin composites depended on both the variety and concentration of nanoclay. In this regard, composites containing MMT displayed higher mechanical strength (both flexural and compression) than those resins prepared with PLG due to a poor nanoclay dispersion as revealed by SEM. Solubility of the composites was dependent not only on nanoclay-type but also the mineral concentration. Dental composites fulfilled the minimum depth cure and solubility criteria set by the ISO 4049 standard. In contrast, the minimum bending strength (50 MPa) established by the international standard was only satisfied by the dental resins containing MMT. Based on these results, composites containing either MMT or PLG (at low filler contents) are potentially suitable for use in dental restorative resins, although those prepared with MMT displayed better results.

## 1. Introduction

Dental resin-based composites (RBCs) for both direct and indirect dental restorations have been in use for the past 50 years [1]. These materials are composed of inorganic particles (conferring most of the mechanical properties to the final material), which are embedded into an organic matrix (consisting of a mixture of crosslinking monomers, a photoinitiator system and other additives, which form a dense cross-linked polymer upon a free-radical copolymerization) [1,2].

Bisphenol A glycidyl methacrylate (Bis-GMA) is the base-monomer most frequently used in the formulations of dental restorative materials since it reduces polymerization shrinkage and enhances both modulus and thermal stability of the resulting materials; however, it also exhibits a high viscosity, which yields usually a heterogeneous material and problems during handling and application of the product. Thus, in order to achieve high filler loading in dental resin composites, low-viscosity diluent monomers such as triethylene glycol dimethacrylate (TEGDMA) are commonly used [3,4,5]. Other monomers have been also used to improve specific properties [1].

Monomers used in the commercial formulations of dental composites have remained largely unchanged, whereas the type, shape, size and distribution of inorganic fillers have undergone significant changes [1]. Recently, the incorporation of filler particles with nanometric dimensions into dental resins has attracted great attention, as it is possible to obtain materials with improved properties with it. Wear resistance, gloss retention, elastic modulus, flexural strength, diametral tensile strength and reduced polymerization shrinkage have been improved by the addition of nanoparticles to dental composites [5,6].

It is worth mentioning that these improvements are generally achieved through addition of a small amount of nanoclay (contrary to what has been observed in conventional inorganic macro- and micro-fillers as they constitute up to 75–85 wt.%). This fact is due to the extremely large interface area provided by the nano-size particles of the suspended filler [7,8,9].

At present, there is an increasing interest in the incorporation of nanoclays into dental composites. Montmorillonite (MMT) is the most commonly used clay, both in its natural form and organically modified. Its use has been reported in Cloisite^®^ Na+ [6,7,9]; Cloisite^®^ 10A [10]; Cloisite^®^ 20A [6] and Cloisite^®^ 93A [7], although Cloisite^®^ 30B is the organoclay most employed [5,6,7,11].

In contrast, there are few studies in the literature involving the use of alternative clay minerals. Tian et al. and Zhang et al. reported the used of palygorskite (attapulgite) in dental resins, while Weidenbach et al. studied materials containing functional halloysite–nanotube filler [12,13,14].

Therefore, the aim of this work is to investigate the influence of nanoclay type at different loading levels (0, 2, 4, 6, 8 and 10 wt.%) on properties of the dental resins composites. Tetraethylene glycol dimethacrylate (TTEGDMA) was used as co-monomer instead of triethylene glycol dimethacrylate (TEGDMA) monomer, as Rüttermann et al. suggested that viscosities of both monomers are similar but the molecular mass of TTEGDMA is higher than TEGDMA; in consequence, it was expected to be more advantageous regarding shrinkage [15]. The TTEGDMA is often used not only in commercial resins but also in experimental dental restorative resins [15,16].

## 2. Materials and Methods

### 2.1. Materials

The monomers used for the preparation of the dental composites were Bisphenol A glycidyl methacrylate (Bis-GMA) and tetraethylene glycol dimethacrylate (TTEGDMA). Camphorquinone (CQ) and *N*,*N*-dimethyl aminoethyl methacrylate (DMAEMA) were used as photo-initiator and co-initiator, respectively. All reagents were purchased from Sigma–Aldrich Co. (Milwaukee, WI, USA) and used as received without further purification. The clay minerals used in this study were a commercial montmorillonite (Cloisite^®^ 30B, abbreviated as MMT) modified with a quaternary ammonium salt (90 meq/100 g of clay) from Southern Clay Products (Gonzáles, TX, USA) and an HCl purified palygorskite (PLG) extracted from a mineral deposit in Chapab, Yucatán, México.

### 2.2. Preparation of Dental Composite

Bis-GMA was mixed manually with TTEGDMA in a glass container at 50:50 wt.% ratio. The photo-initiator CQ and the tertiary amine DMAEMA were then added to the Bis-GMA/TTEGDMA mixture (both materials at 0.5 wt.% to the monomer mixture). The container was covered with aluminum foil (to avoid premature curing) and refrigerated until its use. Monomers and initiator system were manually mixed, and the nanoclay (MMT or PLG) was slowly added at different loading levels in small portions to avoid the formation of agglomerates; formulations were mixed until no filler agglomerations were visually observed in the monomer mixture. Resin composites were cured with a light emitting diode unit (LED H, Woodpecker, Guilin, China) with a wavelength range of 420–480 nm and a light intensity of 1000 mW/cm^2^. Table 1 summarizes the dental resin composites prepared in this study.

### 2.3. Characterization of Dental Composites

#### 2.3.1. Fourier Transform Infrared Spectroscopy (FTIR)

Infrared spectra were obtained with a Thermo Scientific Nicolet 8700 spectrometer (Madison, WI, USA) by the attenuated total reflectance (ATR) technique using a germanium crystal. Samples were analyzed in the 4000 to 650 cm^−1^ wavenumber range with a resolution of 4 cm^−1^ and averaging 100 scans.

#### 2.3.2. Thermogravimetric Analysis (TGA)

Thermogravimetric analysis was performed in a Perkin Elmer TGA-7 thermogravimetric analyzer (Norwalk, CT, USA), from 45 to 750 °C at a heating rate of 10 °C/min, under nitrogen atmosphere. From the first derivative curve, the decomposition temperature of composites was determined.

#### 2.3.3. Dynamic Mechanical Analysis (DMA)

The glass transition temperature (Tg) of composites was determined by dynamic mechanical analysis using a Perkin Elmer DMA-7 (Norwalk, CT, USA) in bending mode. Bars of 30 × 10 × 0.5 mm were heated from 35 to 200 °C at 3 °C/min, under nitrogen atmosphere, using a frequency of 1 Hz.

#### 2.3.4. Mechanical Properties

An overlapping irradiation regime was applied to photo-polymerize the specimens for mechanical properties dependent on size and shape; five overlapped irradiations were employed on both sizes (40 s each irradiation; 400 s in total) to cure bending specimens, while one irradiation was used on both sizes (40 s each irradiation; 80 s in total) in compression specimens. All photo-cured specimens were immersed in distilled water at 37 ± 1 °C until mechanical testing (7 days).

In order to establish the influence of both nanoclay-type and filler content on the mechanical properties of dental resins, flexural tests were carried out according to the ISO 4049 standard [17] while compressive tests were performed according to the ASTM D695 standard [18]. For 3-point bending tests, five rectangular samples (25 mm length, 2 mm width and 2 mm thickness) were tested at 1 mm/min in a Shimadzu Autograph AGS-X (Kyoto, Japan) Universal Testing Machine, using a load cell of 1 kN and a distance between supports of 20 mm. For compression tests, five cylindrical specimens (8mm height and 4mm diameter) were deformed at 1 mm/min in a Shimadzu Autograph AG-I (Kyoto, Japan) Universal Testing Machine employing a load-cell of 5 kN.

Flexural strength (σ, MPa) and modulus (E, MPa) were calculated according to Equations (1) and (2), respectively.

(1) $$\sigma =\frac{3Fl}{2bh^{2}}$$

(2) $$E=\frac{Fl^{3}}{4bh^{3}d}$$

where F is the maximum load recorded (N), l is the span between the supports (mm), b is the width of the specimen (mm), h is the height of the specimen (mm) and d is the deflection at load F (mm).

Compressive strength (σ, MPa) was determined according to Equation (3) and the elastic modulus (E, MPa) was calculated as the slope of the elastic part of the stress–strain curve.

(3) $$\sigma =\frac{4F}{\pi D^{2}}$$

where F is the maximum load recorded (N) and D is the diameter of the specimen (mm).

Fracture surfaces from flexural test samples were analyzed by Scanning Electron Microscopy (SEM) using a JEOL JSM-6360 LV (Akishima, Tokyo, Japan) operated at 20 kV. Samples were adhered on aluminum cylinders using a double-sided tape of copper and coated with a thin layer of gold in a Denton Vacuum Desk-II (Moorestown, NJ, USA) sputter coater system for 1 min prior to examination.

#### 2.3.5. Depth of Cure

Depth of cure (Dc, mm) tests were performed on cylindrical specimens (6 mm height; 4 mm diameter) according to Section 7.10 of the ISO 4049 standard [17]. Three samples of each composite were irradiated during 40 s on one side, and the Dc was calculated according to Equation (4)

(4) $$\mathrm{Dc}=\frac{l}{2}$$

where l is the height of the specimen (mm) after removing the uncured material.

#### 2.3.6. Sorption and Solubility

For water sorption and solubility tests, disc-shaped specimens (1 mm thickness; 15 mm diameter) were used according to Section 7.12 of the ISO 4049 standard [17]. Nine overlapped irradiations of 40 s were applied to specimens on one side (360 s in total). Water sorption (Wsp, µg/mm^3^) and solubility (Wsl, µg/mm^3^) were calculated according to Equations (5) and (6), respectively.

(5) $$\mathrm{Wsp}=\frac{m_{2}-m_{1}}{V}$$

(6) $$\mathrm{Wsl}=\frac{m_{1}-m_{3}}{V}$$

where $m_{1}$ is the mass (µg) of the conditioned specimen, $m_{2}$ is the mass of the specimen (µg) after immersion in distilled water at 37 ± 1 °C for 7 days, $m_{3}$ is the mass of the reconditioned specimen (µg) and V is the volume of the specimen (mm3).

### 2.4. Statistical Analysis

One-way analysis of variance (ANOVA) and Tukey’s test (P < 0.05) were used to determine significant differences between properties of dental resin composites prepared with either MMT or PLG.

## 3. Results and Discussion

### 3.1. Fourier Transform Infrared Spectroscopy (FTIR)

**Figure 1:**
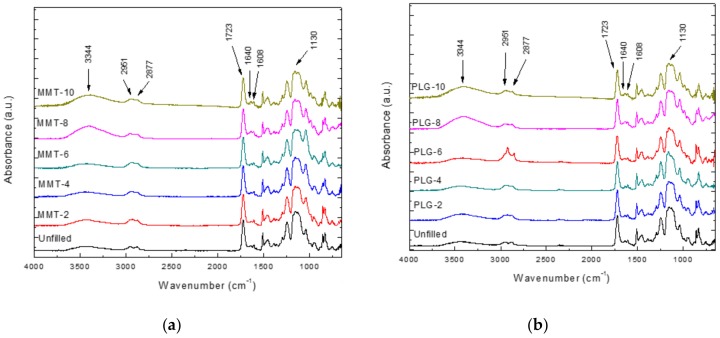
FTIR spectra of nanocomposites prepared with either (**a**) montmorillonite (MMT) or (**b**) palygorskite (PLG).

FTIR spectra of neat resin and its nanocomposites prepared with either MMT or PLG are shown in Figure 1a,b, respectively. As noted, spectra of nanocomposites were very similar to those obtained for pure resin, probably due to the low filler content. Thus, a broad band was observed at 3344 cm^−1^, attributed to O-H stretching vibration of hydroxyls in Bis-GMA structure; bands at 2951 and 2877 cm^−1^ related to asymmetric and symmetric stretching vibrations of the methylene group, and an intense band at 1723 cm^−1^ owing to C=O stretching of ester groups from dimethacrylates (Bis-GMA and TTEGDMA). Spectra also showed bands at 1640 and 1608 cm^−1^ which correspond to the stretching vibration of the aliphatic (from vinyl group of monomers) and aromatic (from benzene ring) C=C bonds, respectively [19]; in fact, the height ratio of these bands is generally used to determine the degree of conversion of dental resins [20]. Finally, an intense band around 1130 cm^−1^ was also detected and associated with symmetric vibration of C-O-C linkage from TTEGDMA structure.

### 3.2. Thermogravimetric Analysis (TGA)

**Figure 2:**
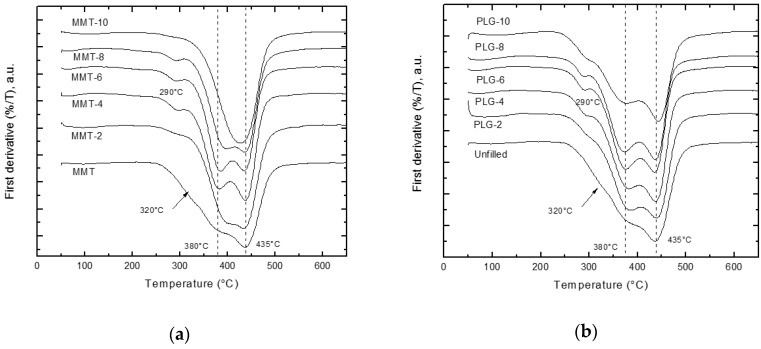
DTGA curves for nanocomposites prepared with either (**a**) MMT or (**b**) PLG.

Figure 2 shows the DTGA (First derivative of the TGA curve) curves for pure resin, obtained from Bis-GMA and TTEGDMA, and its composites prepared with either MMT (Figure 2a) or PLG (Figure 2b). As can be seen, pure resin presented two well-defined degradation stages (Td) at 380 and 435 °C, although a broad transition at lower temperatures (320 °C) was also observed. Teshima et al. studied the thermal degradation of resins prepared from Bis-GMA and TEGDMA and detected three degradation steps during thermal decomposition of this material. They found that methacrylic acid and 2-hydroxyethyl methacrylate are released during the first and second stages, whereas propionic acid and phenol are produced in the final stage [21].

On the other hand, it has been suggested that addition of nanoclays into polymeric matrices could improve the thermal stability of the resulting material [22]. This fact was not observed in nanocomposites prepared in this study; i.e., temperatures observed for dental resin composites were practically similar to those obtained for neat resin. The latter is in agreement with Munhoz et al., who pointed out that it was not possible to identify an impact of the presence of the modified clay on the thermal stability of the composites [23]. However, a close inspection of thermograms allowed detection of small changes in thermal degradation behavior in some composites. For instance, the degradation stage at 380 °C in some MMT nanocomposites was shifted to higher temperatures overlapping with that of 435 °C in composites containing 10 wt.%. This could be associated with interaction between surfactant and crosslinking polymer structures. Mahnoodian et al. studied the thermal degradation of Bis-GMA/TEGDMA/Cloisite 30B nanocomposites and found that the pure resin exhibited two decomposition stages at 292 and 392 °C. The first temperature did not change when MMT was added to resins, although temperature corresponding to the second decomposition step was reduced with increasing clay content [7].

Finally, it should be also mentioned that the broad transition observed at 320 °C was shifted to lower temperatures (290 °C) in nanocomposites, being more evident when nanoclay content was increased. This event could be related to the emission of volatiles such as water and fragments of organic modifier from PLG and MMT nanoclays respectively.

### 3.3. Dynamic Mechanical Analysis (DMA)

Glass transition temperatures of dental composites were obtained from the maximum of the tan d versus temperature curves and their maxima are summarized in Table 2. Thermograms exhibited a one broad peak at around 110 °C, which suggests a homogeneous polymeric network; this value is in agreement with that reported by Terrin et al. for dental resins composed mainly of Bis-GMA and TEGDMA [9]. Composites containing PLG showed slightly higher values than those obtained for the corresponding MMT composites. It is also noted that dental resins prepared with PLG exhibited an increase in this parameter as nanoclay content was increased. In contrast, when MMT was added to dental resin formulations, values decreased slightly from 110 to 108 °C returning to higher Tg values (112 °C) at nanoclay contents of 8 and 10 wt.%; a similar trend was reported by Terrin et al., who studied resin-based composites with organically modified MMT [9]. The shift of the relaxation temperature in composites containing nanoclays towards higher temperatures is attributed to immobilized polymeric chains at the polymer/filler interphase [23].

### 3.4. Mechanical Properties

**Figure 3:**
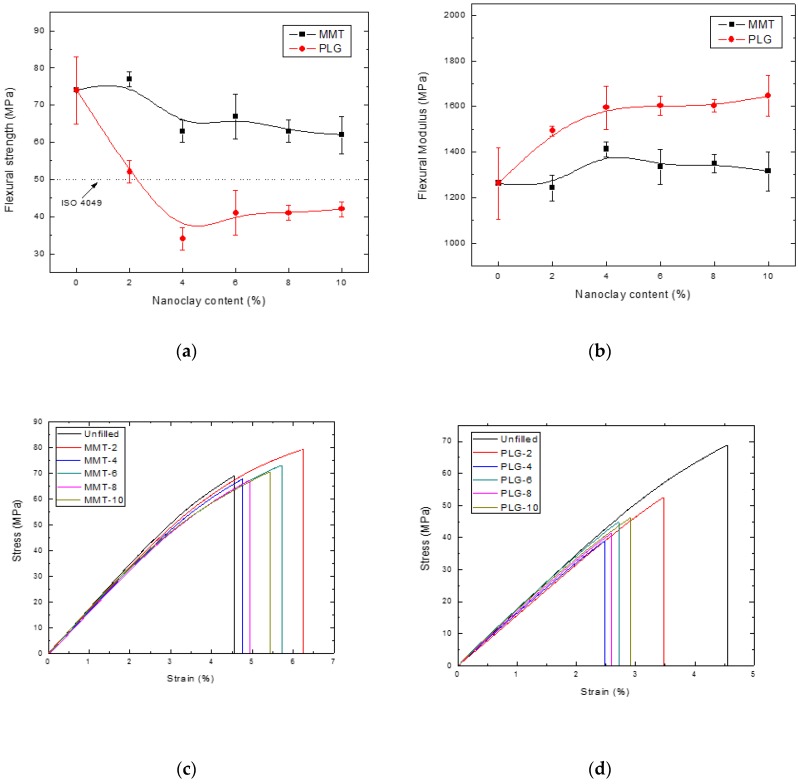
Flexural properties of dental resin composites: (**a**) Strength; (**b**) modulus; (**c**) stress–strain curves for nanocomposites prepared with MMT and (**d**) stress–strain curves for nanocomposites prepared with PLG.

**Figure 4:**
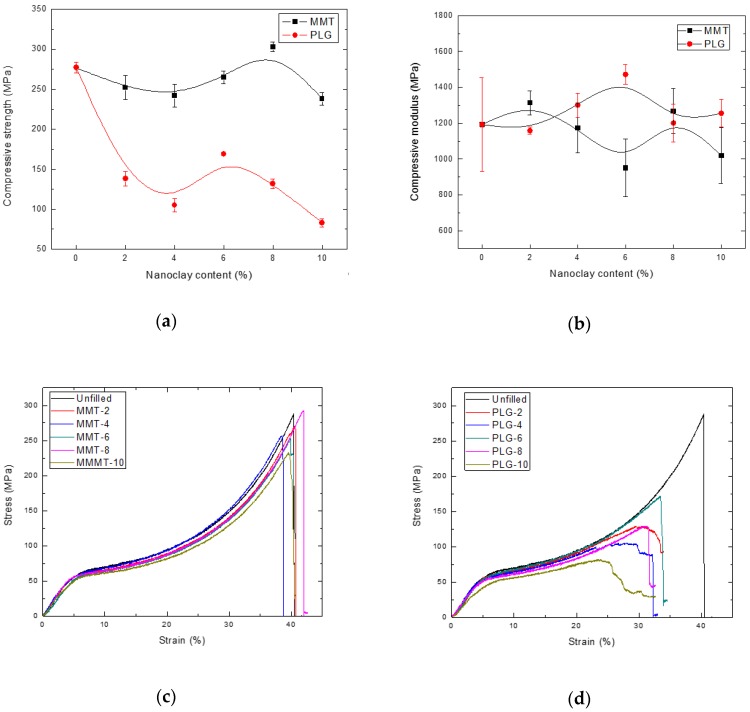
Compressive properties of dental resin composites: (**a**) Strength; (**b**) modulus; (**c**) stress–strain curves for nanocomposites prepared with MMT and (**d**) stress–strain curves for nanocomposites prepared with PLG.

Figure 3 and Figure 4 show the flexural and compressive properties of the nanocomposites prepared in this study, respectively. As can be seen, the mechanical properties of dental resin composites varied depending on the type and concentration of the inorganic aggregate.

Regarding flexural properties (Figure 3), it was observed that strength decreased monotonically with the MMT loading; on the other hand, samples containing PLG exhibited a drastic decrease of strength when the additive was added (i.e., 2 wt.%) and remained almost constant at higher filler concentration. It is worth mentioning that the minimum flexural strength set by the ISO 4049 standard [17] (50 MPa) was fulfilled by all composites containing MMT and that prepared with PLG at 2 wt.%. Typical stress–strain curves for nanocomposites prepared with either MMT or PLG are presented in Figure 3c,d, respectively.

The flexural modulus tended to increase with filler concentration regardless of type of nanoclay employed; MMT composites seemed to exhibit slight increments, but the differences were not statistically significant). PGL composites showed higher modulus with PLG up to 4 wt.% clay content, and no statistical differences were found for composites with higher clay content. Several authors [4,24,25] have also reported that flexural strength of nanocomposites decreased and modulus increased [8,10] as filler content increased.

**Figure 5:**
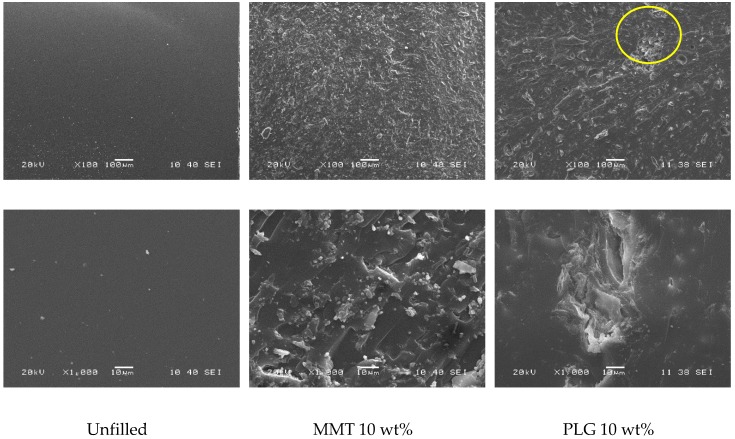
SEM micrographs of fracture surface from bending specimens.

It is well known that the dispersion of nanoparticles and compatibility between phases (filler and matrix) play a key role in the enhancement of mechanical properties of nanocomposites. Thus, it is probable that MMT presented better results than PLG as it was organically modified to improve its interaction with organic polymers and increase the interlayer spacing favoring its dispersion, as observed in the SEM analysis (see Figure 5).

Compressive properties (Figure 4) were not greatly affected with addition of nanoclay content, except compressive strength of materials containing PLG; these properties decreased with nanoclay concentration as reported by Mucci et al. [8]. Interestingly, the strength of composites containing MMT also showed higher values than corresponding PLG materials. Typical stress–strain curves for nanocomposites prepared with MMT or PLG are presented in Figure 4c,d, respectively.

Figure 5 shows the nanoclay dispersion within the dental composites. Unloaded dental resin exhibited a clear brittle fracture producing a smooth surface; the presence of nanoclays produced a different fracture mechanism generating a rough surface upon breaking. SEM observation at higher magnification revealed that nanoclays were dispersed differently within dental resin; MMT seems to be better distributed in the resin due to its surface modification than PLG, which was distributed as agglomerates.

### 3.5. Depth of Cure

Higher curing depth of restorative materials is one of the factors that determine the quality of the material [26]. Curing depth results obtained for dental composites prepared with either MMT or PLG are displayed in Table 3. It can be seen that the curing depth was not affected either by the nanoclay-type or filler content. Further, all composites exhibited higher values than that established in the ISO 4049 standard [17] (1.5 mm), and even better values than those reported by other authors [26].

### 3.6. Sorption and Solubility

**Figure 6:**
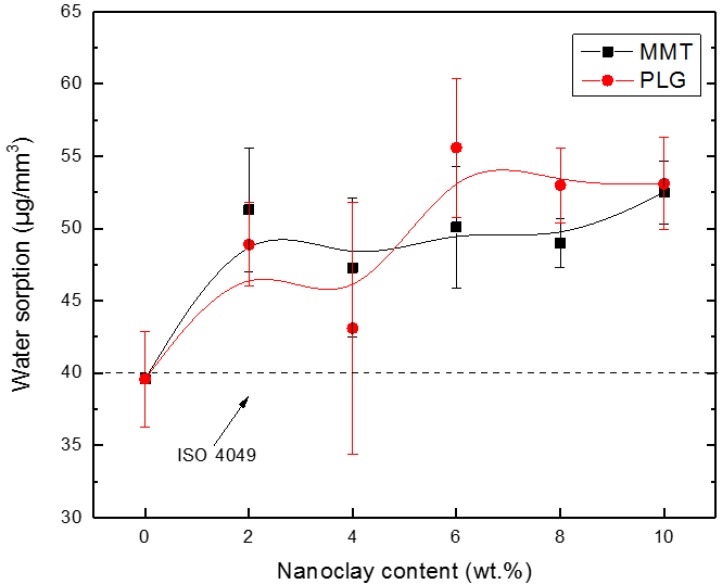
Water sorption of dental composites prepared with either MMT or PLG at several concentrations.

The water sorption for neat resin and its composites prepared with either MMT or PLG at different contents are shown in Figure 6. As expected, results showed that clays induce water sorption in the composites (although this parameter seems not to be affected by the nanoclay type) as it is well known that natural clays have a hydrophilic character and are naturally prone to absorbing water [23].

The hydrophilic behavior of composites depends on characteristics of constituents, i.e., organic matrix and inorganic filler. In this regard, unfilled resin exhibited a water absorption slightly below 40 mg/mm^3^ (which is the maximum value for dental restorative materials stated by ISO 4049), whereas all nanocomposites exhibited water sorption values slightly higher.

Interestingly, composites containing PLG exhibited a similar water sorption behavior to that displayed by materials prepared with MMT, although the latter clay was modified organically by a cation exchange reaction between the silicate and methyl, tallow, bis-2-hydroxyethyl and quaternary ammonium chloride in order to reduce the clay hydrophilicity. This could be attributed to the presence of two hydroxyethyl groups in the organoclay as suggested by Mucci et al. [8].

Water uptake in dental resin composites occurs by diffusion of water molecules within a polymeric matrix and may cause hydrolytic degradation of the matrix and/or filler matrix interface [23], yielding leachable substances, which could be quantified in a solubility test.

**Figure 7:**
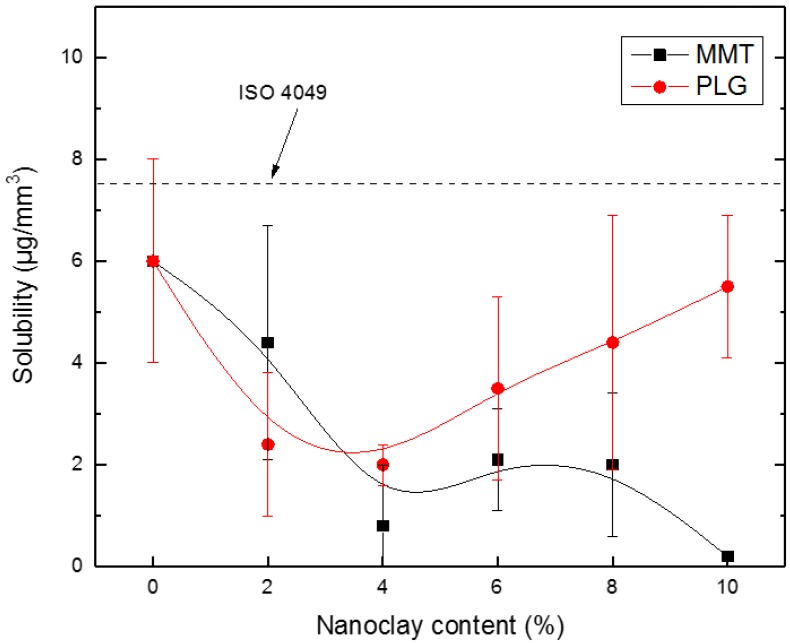
Solubility of dental composites prepared with either MMT or PLG at several concentrations.

Figure 7 shows the solubility results obtained from composites prepared with either MMT or PLG at several concentrations. It is clear that materials containing palygorskite exhibit a different behavior than that displayed by MMT composites. For instance, when PLG was added to dental resin, solubility decreased at lower nanoclay contents and then practically returned to the initial value at higher PLG concentrations; in contrast, solubility of composites containing MMT decreased monotonically with increasing nanoclay content. Regardless of the above fact, a statistically significant difference was only detected between nanocomposites containing 10 wt.% of clay. Further, it is interesting to note that solubility measurements for all composites (including an unfilled sample) remained below 7.5 mg/mm^3^ as suggested by the ISO 4049 [17] standard for dentistry-polymer-based restorative materials.

## 4. Conclusions

Bis-GMA/TTEGDMA-based resin composites with two different types of nanoclays were successfully prepared and characterized. Results indicate that Tg, Td, depth of cure and water absorption were not greatly affected by the type of nanoclay, while the mechanical properties of dental resin composites depended on nanoclay type and concentration of inorganic filler. In general, MMT composites displayed higher mechanical strength than those shown by resins prepared with PLG, due to dispersion problems as revealed by SEM. Solubility of the composites was also dependent on nanoclay type and the mineral concentration. In general, dental composites prepared in this study fulfilled the minimum depth cure and solubility criteria set by the ISO 4049 standard. In contrast, the minimum bending strength (50 MPa) established by the international standard was only satisfied by dental resins containing MMT. Based on these results, composites containing either MMT or PLG (at low filler contents) are potentially suitable for use in dental restorative resins, although those prepared with MMT displayed better results.

## Figures and Tables

**Table 1:** Composition of dental composites.

Nanoclay Content (wt.%)	Composites	
	MMT	PLG
0	Unfilled	
2	MMT-2	PLG-2
4	MMT-4	PLG-4
6	MMT-6	PLG-6
8	MMT-8	PLG-8
10	MMT-10	PLG-10

**Table 2:** Glass transition temperatures (Tg) of dental composites.

Nanoclay Content (%)	Tg (°C)	
	MMT	PLG
0	110	
2	108	111
4	110	112
6	110	116
8	112	116
10	112	116

**Table 3:** Depth of cure of dental composites.

Nanoclay Content (%)	Depth of cure (mm)	
	MMT	PLG
0	3.0 + 0.01	
2	2.99 + 0.01	2.99 + 0.02
4	2.99 + 0.01	2.99 + 0.01
6	2.99 + 0.02	2.99 + 0.02
8	2.99 + 0.03	2.99 + 0.02
10	2.99 + 0.04	2.99 + 0.05
